# Sphingolipid-associated signature unveils TIMP1-driven temozolomide resistance and guides stratified therapy in glioblastoma

**DOI:** 10.3389/fimmu.2026.1753274

**Published:** 2026-03-18

**Authors:** Feng Lyu, Jingjing Wu, Ji Qi, Gege Wang, Liqing Xie, Zhicong Wang

**Affiliations:** 1 Department of Radiology, The First Affiliated Hospital of Xiamen University, School of Medicine, Xiamen University, Xiamen, China; 2 German Center for Neurodegenerative Diseases (DZNE), Medical Faculty, University of Bonn, Bonn, Germany; 3 Department of Neurosurgery, Guangdong Provincial People’s Hospital, Guangdong Academy of Medical Sciences, Southern Medical University, Guangzhou, China; 4 Institute of Nervous System Diseases, Xuzhou Medical University, Xuzhou, Jiangsu, China; 5 Department of Neurology, The Affiliated Hospital of Xuzhou Medical University, Xuzhou, Jiangsu, China; 6 Department of Child Healthcare, Children's Hospital of Fudan University (Xiamen Branch), Xiamen Children's Hospital, Xiamen, China

**Keywords:** glioblastoma, pharmacogenomics, prognostic model, sphingolipid metabolism, temozolomide resistance, TIMP1, tumor microenvironment

## Abstract

**Background:**

Glioblastoma (GBM) remains the most prevalent and aggressive primary central nervous system (CNS) malignancy; however, the clinical efficacy of the preferred chemotherapeutic agent, Temozolomide (TMZ), is severely compromised by innate and acquired resistance. Sphingolipid metabolism acts as a pivotal regulator of GBM cell fate, and the imbalance of the “sphingolipid rheostat” is intimately linked to TMZ resistance. This provides potential targets for developing novel prognostic models to inform stratified treatment risk strategies, while offering a promising entry point for TMZ chemosensitization and stratified drug combinations.

**Methods:**

We integrated single-cell and bulk transcriptomics from TCGA and GEO. Through a multi-dimensional framework combining Weighted Gene Co-expression Network Analysis (WGCNA), differential expression profiling, Cox regression, and machine learning, we identified candidate genes associated with the molecular landscape coupled with sphingolipid dysregulation and TMZ sensitivity in GBM to construct a reliable prognostic model. We verified mRNA expression of model genes and protein expression of TIMP1 in clinical specimens via RT-qPCR and tissue microarrays (TMA), respectively. Furthermore, we functionally characterized the core target, TIMP1, via lentiviral knockdown in U87 cells, employing Transwell, CCK-8, and IC50 assays to evaluate its impact on malignancy and, crucially, its capacity to modulate TMZ chemosensitization.

**Results:**

Single-cell analysis stratified GBM samples into distinct metabolic subclasses, revealing significant metabolic heterogeneity. Integrating TCGA and GEO profiles with WGCNA-based multi-dimensional intersection, we identified 95 candidate genes, refined via Cox regression and machine learning into a potent six-gene model (MXRA8, TIMP1, TREM1, S100A4, RMI2, IRF7) reflecting critical axes of extracellular matrix (ECM) remodeling, inflammation, and DNA repair. We delineated the model’s role in shaping an immune-excluded tumor microenvironment (TME) characterized by stromal remodeling, T-cell exhaustion and functional impairment of natural killer (NK) cell subsets, while uncovering specific therapeutic vulnerabilities for distinct risk subgroups. Experimental validation confirmed widespread upregulation of core targets in clinical specimens. Functionally, TIMP1 knockdown significantly suppressed proliferation and invasion. Most importantly, silencing TIMP1 effectively restored sensitivity to TMZ (chemosensitization).

**Conclusions:**

This study establishes and validates a robust GBM prognostic model integrating the sphingolipid-associated molecular landscape with chemotherapy resistance. It provides a comprehensive perspective on the interplay among sphingolipid dysregulation, immune evasion, TMZ resistance, and the critical functional role of TIMP1. Beyond enabling precise patient stratification, this model highlights specific therapeutic vulnerabilities, offering a translational framework for developing combinatorial strategies to target the sphingolipid regulatory network and overcome GBM chemoresistance.

## 1 Introduction

The clinical management of GBM, the most aggressive primary intracranial malignancy, remains deadlocked by inevitable recurrence and therapeutic resistance under current standard-of-care regimens (1). Despite TMZ functioning as the systemic cornerstone, its efficacy is severely compromised by intrinsic and acquired resistance mechanisms (2). These mechanisms, including the repair of cytotoxic DNA damage induced by the alkylating activity of TMZ (3), engender profound heterogeneity in patient survival. This persistent clinical bottleneck underscores a critical deficit: the absence of reliable, mechanism-based multigenic signatures to accurately stratify patient risk and guide precision intervention. Consequently, there is an urgent imperative to decipher the molecular underpinnings of TMZ resistance to engineer novel prognostic models, identify actionable targets, and screen for candidate agents for stratified chemosensitization.

Sphingolipid metabolism emerges as a pivotal metabolic nexus governing cell fate and therapeutic response in GBM. The “sphingolipid rheostat,” a dynamic equilibrium between pro-apoptotic ceramide and pro-survival sphingosine-1-phosphate (S1P), is frequently hijacked by tumor cells to evade cytotoxicity (4). Although standard therapies strive to shift this rheostat toward apoptosis (5), metabolic rewiring in GBM often sustains a pro-survival phenotype, thereby fostering a permissive niche for drug resistance and shaping an immune-excluded and potentially exhausted TME (6). Importantly, this dysregulation of sphingolipid metabolism may extend beyond intrinsic cellular resistance to potentially drive downstream stromal remodeling. This pathological ECM reorganization is thought to construct a physical barrier that restricts drug delivery and spatially limits immune cell infiltration. Despite this compelling mechanistic link, the clinical translation of this sphingolipid metabolic landscape into effective prognostic tools and stratified therapeutic strategies remains largely unrealized.

Here, we investigate the sphingolipid-associated molecular landscape and TMZ resistance by integrating single-cell and bulk transcriptomic datasets. Utilizing a rigorous single-cell and bulk transcriptomic framework, our analysis unveiled a distinct set of GBM-specific Sphingolipid-associated TMZ Resistance (GSTR) genes, from which we distilled a potent six-gene prognostic model validated across independent cohorts. Beyond risk stratification, we delineated the role of this six-gene model in shaping the TME and uncovered distinct therapeutic vulnerabilities specific to different risk subgroups. Furthermore, we demonstrated the widespread upregulation of the model’s core genes in clinical GBM tissues. Critically, functional validation revealed that silencing the representative gene TIMP1 not only suppresses malignant phenotypes but effectively restores TMZ sensitivity. This study thus establishes a novel, mechanism-based framework for precision prognosis and provides a compelling rationale for targeting the sphingolipid axis to overcome chemotherapy resistance in GBM.

## 2 Materials and methods

### 2.1 Data acquisition and preprocessing

Transcriptomic profiles and corresponding clinical data for GBM were retrieved from The Cancer Genome Atlas (TCGA) database (https://portal.gdc.cancer.gov/). As of the time of manuscript preparation, this cohort comprised 170 GBM samples, including 154 samples with complete survival information, and 5 control samples. Normal brain tissue data were supplemented by the Genotype-Tissue Expression (GTEx) database (https://gtexportal.org/). Datasets GSE100736, GSE173278, and GSE74187 were obtained from the Gene Expression Omnibus (GEO) (https://www.ncbi.nlm.nih.gov/geo/), while the CGGA-325 dataset was retrieved from the Chinese Glioma Genome Atlas (CGGA) (http://www.cgga.org.cn/). In detail, GSE100736 contained transcriptomes from paired TMZ-sensitive and -resistant samples (n=3 each), and GSE173278 offered single-cell RNA sequencing (scRNA-seq) files for 10 primary isocitrate dehydrogenase (IDH)-wildtype GBM tumors. Both GSE74187 and CGGA-325 were utilized for external validation purposes. Additionally, a comprehensive set of 97 sphingolipid metabolism-related genes (SMRGs) was curated from the InnateDB portal (http://www.innatedb.com) (7).

### 2.2 Single-cell transcriptome analysis

We utilized the Seurat R package (version 4.3.0) to process single-cell transcriptomic files from GSE173278 (8). For quality control, cells exhibiting a mitochondrial gene content >10% were excluded via the PercentageFeatureSet function. We applied the NormalizeData function to standardize the expression matrix, after which the top 2,000 highly variable genes were determined via FindVariableFeatures. To eliminate batch effects across multiple samples, canonical correlation analysis (CCA) was employed. Principal component analysis (PCA) was then executed, with the optimal number of principal components determined via the elbow plot technique. We accomplished cell clustering through the FindNeighbors and FindClusters functions within Seurat. To detect marker genes for specific clusters, we applied FindAllMarkers with specific criteria: min.pct = 0.6, only.pos = TRUE, and logfc.threshold = 0.5. Cell subtypes were annotated by mapping these markers against the CellMarker database, and annotations were subsequently corroborated by the SingleR algorithm. Furthermore, GBM samples were scored based on SMRG expression profiles using AUCell (version 1.20.2) (9), allowing for stratification into high and low sphingolipid metabolism (SM) groups based on the median score. Finally, differentially expressed genes (DEGs) between the high and low SM groups were identified using the limma R package (version 3.54.2) (10) (adjusted P < 0.05). Functional differences between these groups were explored via Gene Set Variation Analysis (GSVA) (version 1.46.0) (11) using the “h.all.v2023.1.Hs.symbols.gmt” gene set.

### 2.3 Sphingolipid metabolism stratification and weighted gene co-expression network analysis

Leveraging SMRG expression profiles within the TCGA-GBM cohort, we computed SM scores through GSVA (11), subsequently stratifying patients into high and low SM score subgroups using an optimal threshold. Survival disparities were assessed via Kaplan-Meier (K-M) curves and confirmed in the external dataset GSE74187. To detect critical gene modules, we applied the WGCNA R package (version 1.70-3) to the TCGA-GBM dataset (12). Clustering analysis was utilized to screen out outlier samples. An appropriate soft-thresholding power was determined to guarantee a scale-free network topology. An adjacency matrix was constructed to derive the topological overlap measure (TOM), followed by hierarchical clustering dendrogram generation. Modules were identified using the dynamic tree cut algorithm. Key modules were defined as those exhibiting a significant correlation with the SM score (P < 0.05 and |Correlation| ≥ 0.3).

### 2.4 Identification of GSTR genes

To identify candidate genes, we performed a multi-dimensional intersection analysis. First, DEGs between GBM and control samples in TCGA-GBM (|log2FC| ≥ 0.5, P < 0.05) and DEGs between TMZ-resistant and TMZ-sensitive samples in GSE100736 (|log2FC| ≥ 0.5, adjusted P < 0.05) were identified using DESeq2 (version 1.36.0) (13). We intersected upregulated genes from both datasets to define TMZ-related upregulated genes, and similarly for downregulated genes. Subsequently, these TMZ-related DEGs were intersected with DEGs derived from the high/low SM stratification (Section 2.2) and genes from the key WGCNA modules (Section 2.3). This intersection yielded the final set of GSTR genes. Functional enrichment analyses, including Gene Ontology (GO) and Kyoto Encyclopedia of Genes and Genomes (KEGG), were performed using clusterProfiler (version 4.4.4) (14) and org.Hs.eg.db (version 3.12.0).

### 2.5 Construction and validation of the prognostic model

We evaluated the prognostic value of GSTR genes by running univariate Cox regression via the survival R package (version 3.3-1), retaining only those candidates with a P < 0.05. To narrow down these candidates, we utilized the Least Absolute Shrinkage and Selection Operator (LASSO) algorithm implemented in the glmnet package (version 4.1-6) (15). Simultaneously, the Support Vector Machine-Recursive Feature Elimination (SVM-RFE) algorithm was applied to rank genes based on importance, selecting the subset with the lowest error rate. Genes overlapping between the LASSO and SVM-RFE outputs were entered into multivariate Cox regression to establish the definitive six-gene prognostic model. Risk scores were quantified using the equation: Risk Score = α1×X1+α2×X2+…+αn×Xn, where α represents the regression coefficient, and X indicates the gene expression level. The median risk score served as the threshold to categorize patients into high- and low-risk subgroups. We examined survival disparities through K-M analysis and gauged predictive precision using Receiver Operating Characteristic (ROC) curves. Validation of the model was conducted within the CGGA-325 dataset.

### 2.6 Independent prognostic analysis and functional enrichment

Clinical characteristics (age, gender, IDH status, MGMT status, subtype, Karnofsky Performance Status (KPS)) were compared between risk groups using the Chi-square test. Univariate and multivariate Cox regression analyses were conducted to identify independent prognostic factors (P < 0.05), following verification of the proportional hazards (PH) assumption. Subsequently, we generated a prognostic nomogram via the rms package (version 6.2-0) (16), verifying its accuracy through calibration plots and ROC curves. Finally, Gene Set Enrichment Analysis (GSEA) was implemented to uncover biological pathways associated with the high- and low-risk classifications (Reference set: c2.cp.kegg.v7.4.symbols.gmt).

### 2.7 Immune microenvironment profiling

The ESTIMATE algorithm (version 1.0.13) (17) was used to calculate Immune, Stromal, and ESTIMATE scores, which were compared between risk groups using the Wilcoxon rank-sum test. Immune cell infiltration was quantified using the CIBERSORT algorithm. The expression of immune checkpoint genes (18) was analyzed across risk groups. Additionally, Tumor Immune Dysfunction and Exclusion (TIDE) scores were obtained (http://tide.dfci.harvard.edu/) to assess potential responses to immunotherapy, analyzing correlations between TIDE scores and risk scores within the TME. Subsequently, a transcriptional T-cell exhaustion score was computed at the sample level using single-sample gene set enrichment analysis (ssGSEA) implemented in the GSVA framework, based on a representative T-cell exhaustion signature retrieved from the MSigDB database. Finally, to approximate NK functional state, we calculated a cytotoxicity-to-inhibitory ratio per sample by summarizing the expression of an NK cytotoxic effector module relative to an NK inhibitory receptor module; this ratio was used for group comparisons together with the expression of key NK effector molecules and receptors.

### 2.8 Drug sensitivity analysis

Drug sensitivity for 138 chemotherapeutic agents was predicted using the Genomics of Drug Sensitivity in Cancer (GDSC) database. IC50 values were estimated and correlated with risk scores to identify agents with differential efficacy between risk groups.

### 2.9 Cell culture and reagents

U-87 MG, a human GBM cell line, was acquired from GeneChem (Shanghai, China). Maintenance was performed in MEM enriched with 10% fetal bovine serum (FBS) under standard conditions (37 °C, humidified 5% CO_2_ atmosphere). For drug sensitivity assays, cells were treated with various concentrations of TMZ (Solarbio, Beijing, China; Cat. No. IT1330).

### 2.10 Antibodies, lentiviral transduction, and stable cell line establishment

Primary antibodies included anti-TIMP1 (Proteintech, Cat. No. 30869-1-AP; 1:1000 dilution) and anti-GAPDH (Santa Cruz Biotechnology, Cat. No. sc-32233; 1:8000 dilution). Secondary antibodies were HRP-linked anti-rabbit IgG (CST, Cat. No. #7074; 1:10000) and HRP-linked anti-mouse IgG (CST, Cat. No. #7076; 1:10000). For stable gene silencing, lentiviral vectors targeting TIMP1 were constructed by Shanghai GeneChem Co., Ltd. Four vectors were utilized: a negative control (NC, CON313) and three TIMP1-targeting RNAi constructs (KD1: PSC73511-11; KD2: PSC73512-1; KD3: PSC73513-1). The target sequences were: KD1 (5′-GCACAGTGTTTCCCTGTTTAT-3′), KD2 (5′-CCAGCGTTATGAGATCAAGAT-3′), and KD3 (5′-CTGTTGTTGCTGTGGCTGATA-3′). U-87 MG cells were seeded in 6-well plates and infected with the aforementioned lentiviruses at a multiplicity of infection (MOI) of 5, supplemented with HiTransG A infection reagent. The culture medium was replaced 16 h post-infection. At 72 h post-infection, stable transfectants were selected using 2.00 μg/ml Puromycin for 48 h, followed by maintenance in medium containing 1.00 μg/ml Puromycin.

### 2.11 RNA extraction and quantitative real-time PCR

Total RNA was extracted using TRIzol reagent (Shanghai Pufei, Cat. No. 3101-400). cDNA synthesis was performed using the PrimeScript RT Master Mix (Takara, Cat. No. RR036A). RT-qPCR was conducted using SYBR Premix Ex Taq (Takara, Cat. No. RR420A) following the manufacturer’s protocol. Relative mRNA expression was normalized to β-actin using the 2^−ΔΔCt method. The clinical cohort included 19 normal non-tumor brain tissues and 16 GBM tissues.

Primers were synthesized by Sangon Biotech (Shanghai, China) with the following sequences:

TIMP1 (Pair 1): Forward 5′-ATTCCGACCTCGTCATCAGG-3′,

Reverse 5′-GCATCCCCTAAGGCTTGGAA-3′;

TIMP1 (Pair 2): Forward 5′-CTTCTGGCATCCTGTTGTTG-3′,

Reverse 5′-CGCTGGTATAAGGTGGTCTG-3′;

MXRA8: Forward 5′-AACTGGAAGGCAGAGACTTAGA-3′,

Reverse 5′-CTGCAGAAGCACAAGTTTCCA-3′;

S100A4: Forward 5′-GCTTCTTCTTTCTTGGTTTGATCCT-3′,

Reverse 5′-ACTTGTCACCCTCTTTGCCC-3′;

RMI2: Forward 5′-ATGCAGGGCAGGGTAGTGAT-3′,

Reverse 5′-ACCATCACATACTTTCCTGGGAC-3′;

IRF7: Forward 5′-TGTGCTGGCGAGAAGGC-3′,

Reverse 5′-TGGAGTCCAGCATGTGTGTG-3′;

TREM1: Forward 5′-TGTGATCTACCAGCCTCCCA-3′,

Reverse 5′-GGGGTCCCTGAAAAACCCTT-3′;

β-actin: Primer Cat. No. B661102.

### 2.12 TMA and immunohistochemistry

The glioma TMA (Cat. No. NCT805) containing 35 GBM and 5 normal brain tissue samples (duplicate cores per case) was purchased from YEPCOME Biotechnology (Shanghai, China). IHC staining was performed according to standard protocols. TIMP1 protein expression was quantified using QuPath software by measuring the “Smoothed: 25 μm: Cytoplasm: DAB OD mean,” with manual verification to ensure accuracy.

### 2.13 Transwell, Western blotting, CCK-8, and IC50 assays

Transwell migration/invasion assays, Western blotting, Cell Counting Kit-8 (CCK-8) proliferation assays, and IC50 determination were performed as described in previous studies (19, 20).

### 2.14 Statistical analysis

Bioinformatics analyses were conducted using R software (version 4.1.2). Experimental data were analyzed using GraphPad Prism 8.0 (San Diego, CA, USA). Quantitative data are presented as the mean ± standard deviation (SD) or standard error of the mean (SEM) from at least three independent experiments. Differences between two groups were assessed using the two-tailed Student’s t-test or Wilcoxon rank-sum test, while multi-group comparisons utilized one-way ANOVA with Tukey’s *post-hoc* test. Categorical variables were analyzed using the Chi-square or Fisher’s exact test. Correlations were evaluated using Pearson or Spearman coefficients. Survival was analyzed via K-M curves with the Log-rank test. All statistical tests were two-tailed, with P < 0.05 considered statistically significant.

## 3 Results

### 3.1 Identification of cell subpopulations and metabolic heterogeneity in GBM

**Figure 1:**
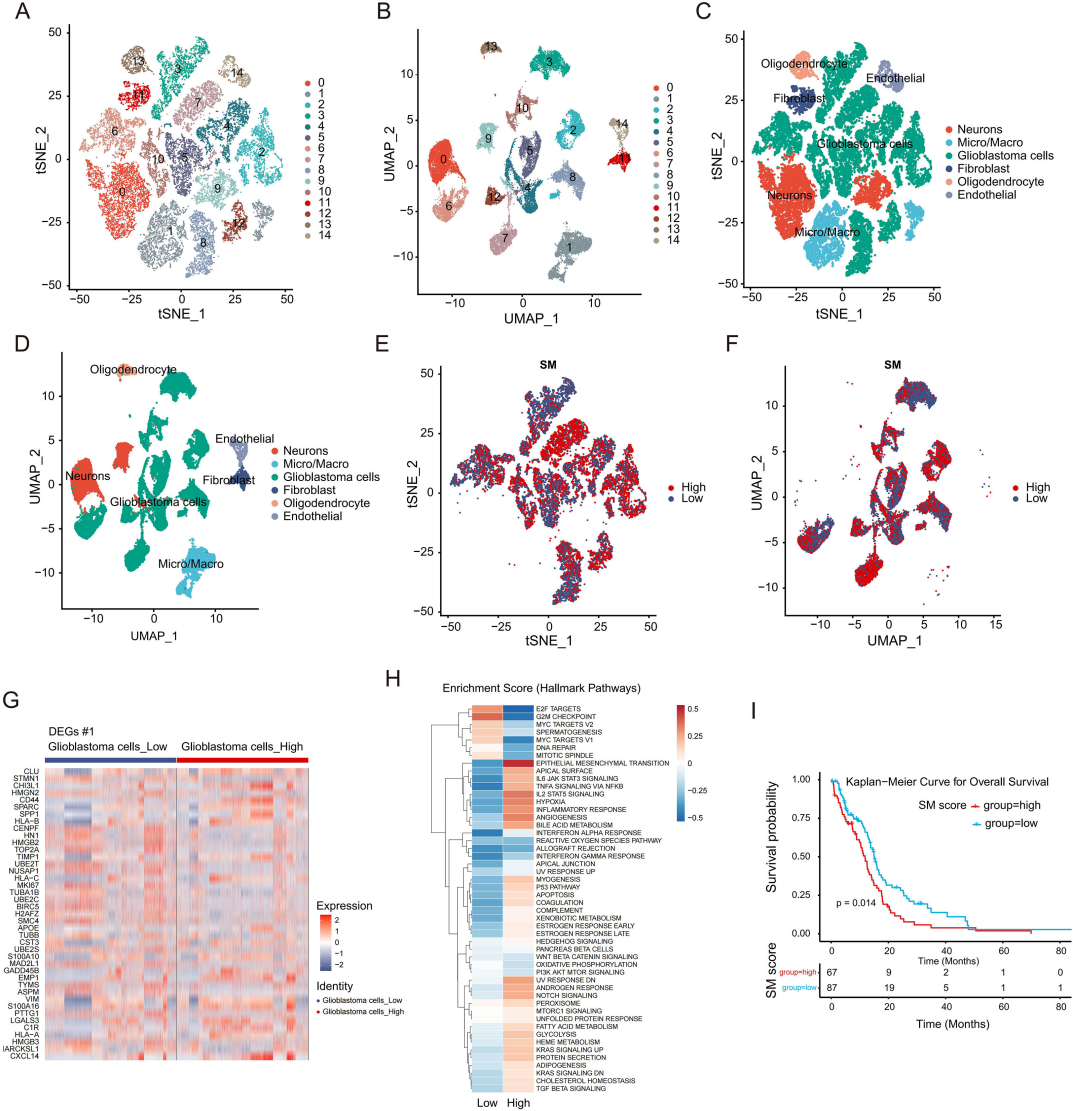
Single-cell transcriptomic landscape and metabolic heterogeneity in GBM. **(A, B)** t-SNE **(A)** and UMAP **(B)** projections identifying 15 distinct cell clusters from scRNA-seq data. **(C, D)** Annotation of six major cell subpopulations within the TME visualized via t-SNE **(C)** and UMAP **(D)**. **(E, F)** Stratification of GBM cells into High (red) and Low (blue) SM groups based on AUCell scoring of SMRGs. **(G)** Heatmap of the top 50 DEGs distinguishing High and Low SM groups. **(H)** GSVA heatmap illustrating differential pathway enrichment between metabolic subgroups. **(I)** K-M analysis of OS for patients stratified by SM score in the TCGA-GBM cohort.

To dissect the cellular heterogeneity of GBM, we analyzed single-cell transcriptomic data. Stringent quality control based on library size, feature counts, and mitochondrial abundance ensured the high fidelity of the dataset (**Supplementary Figure 1A**). We identified the top 2,000 highly variable genes among cells (**Supplementary Figure 1B**), and PCA selected the top 30 principal components for downstream analysis (**Supplementary Figure 1C**). Consensus clustering analysis yielded 15 distinct clusters (**Figures 1A, B**). Referencing canonical markers from previous studies (21), these clusters were annotated into six major cell subpopulations: Neurons, Microglia/Macrophages (Micro/Macr), GBM cells, Fibroblasts, Oligodendrocytes, and Endothelial cells (**Figures 1C, D**; **Supplementary Figure 1D**). Furthermore, by quantifying the activity of 97 SMRGs using AUCell (22), we stratified GBM cells into high and low SM subgroups. This analysis unveiled distinct metabolic heterogeneity within the tumor cell population (**Figures 1E, F**).

### 3.2 Key gene modules correlate with sphingolipid metabolism and prognosis

**Figure 2:**
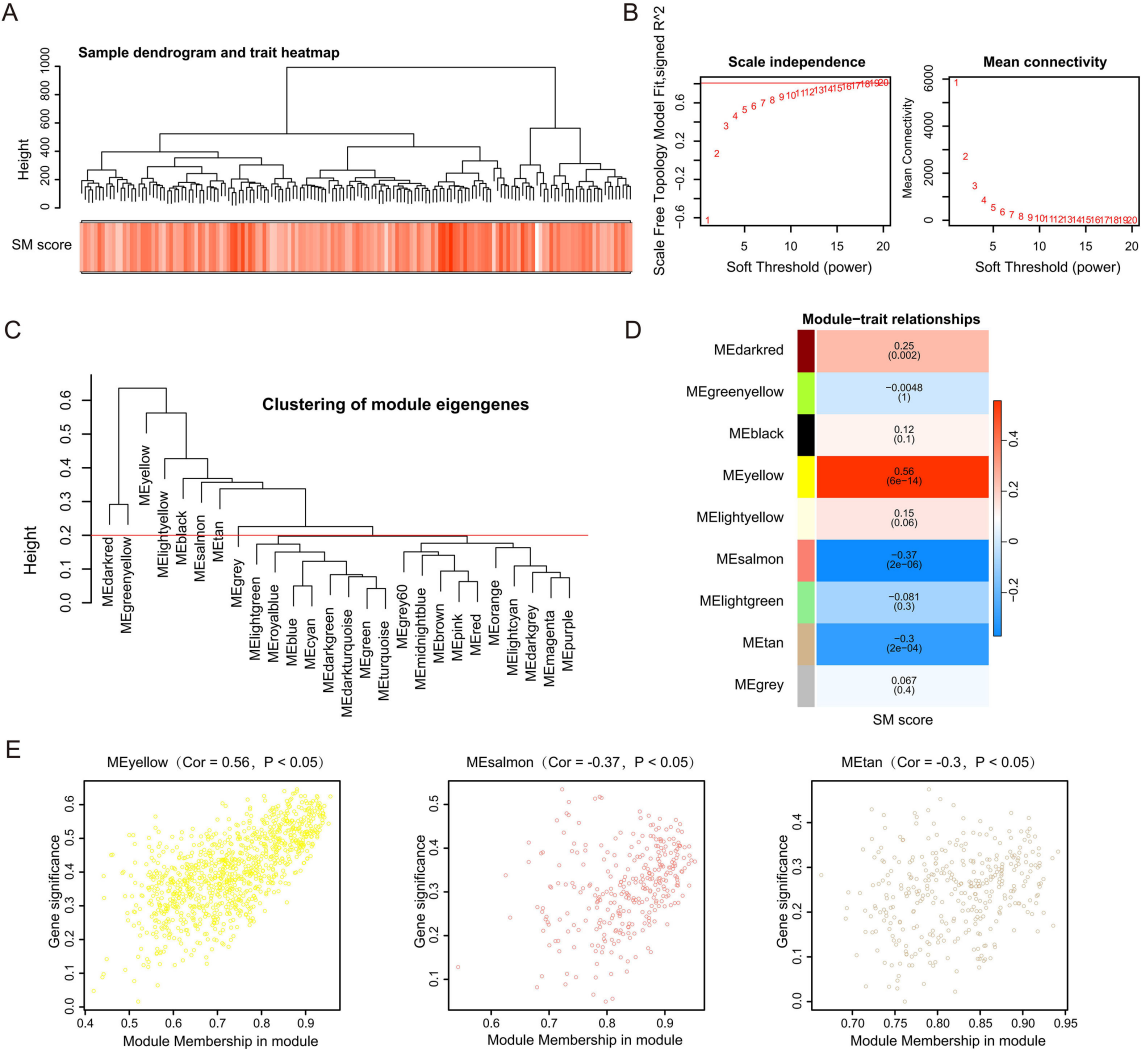
WGCNA identification of key modules linked to sphingolipid metabolism. **(A)** Clustering dendrogram and trait heatmap of samples based on SM scores. **(B)** Determination of the optimal soft-thresholding power (β = 20) showing scale independence and mean connectivity. **(C)** Clustering dendrogram of SMRGs based on topological overlap. **(D)** Module–trait relationship heatmap correlating identified modules with SM scores. **(E)** Gene significance analysis within the key modules.

Having established metabolic heterogeneity, we next sought to explore the molecular features underlying these differences. Differential expression analysis identified 7,158 DEGs distinguishing the high SM group from the low SM group, designated as DEGs #1. (**Figure 1G**). GSVA identified that the high SM group was significantly enriched in a cluster of malignancy-associated pathways, specifically epithelial-mesenchymal transition (EMT), TNF-alpha signaling via NF-kappaB, and hypoxia (**Figure 1H**). Survival analysis indicated significant differences in outcomes, with the high SM group showing markedly poorer survival compared to the low SM group in both the TCGA-GBM and GSE74187 cohorts (**Figure 1I**, **Supplementary Figure 1E**). Subsequently, WGCNA was performed on the TCGA-GBM dataset (**Figure 2A**). An optimal soft-thresholding power of 20 was determined to ensure a scale-free network distribution (**Figure 2B**). Nine co-expression modules were identified (**Figures 2C, D**). Among them, the MEtan (Cor = -0.3, P < 0.05), MEyellow (Cor = 0.56, P < 0.05), and MEsalmon (Cor = -0.37, P < 0.05) modules satisfied the criteria (|Cor| ≥ 0.3, P < 0.05) and were defined as key modules associated with SM scores, collectively containing 1,693 genes (**Figures 2D, E**).

### 3.3 Identification and functional analysis of GSTR genes

**Figure 3:**
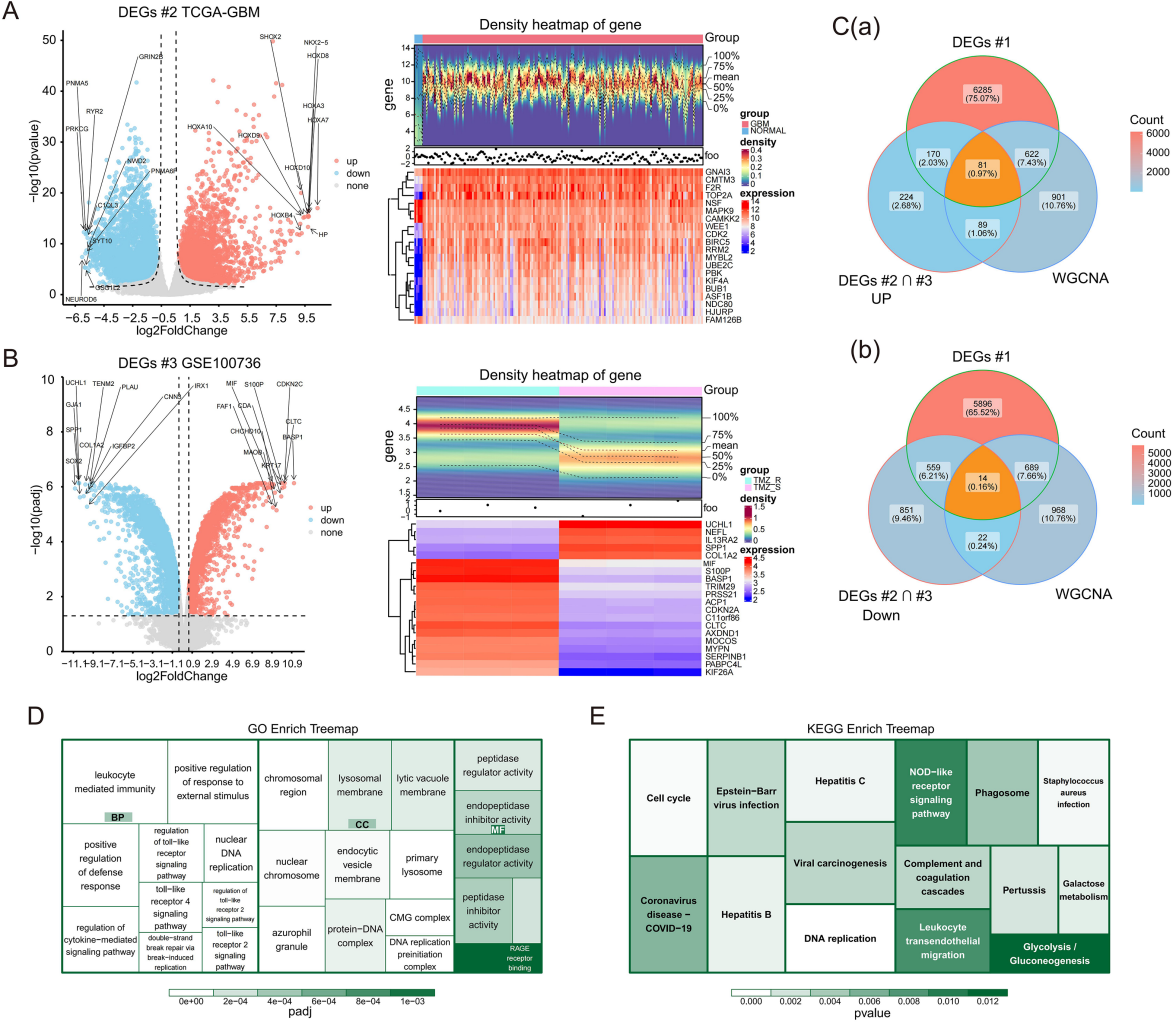
Integrated screening and functional annotation of GSTR genes. **(A)** Volcano plot and heatmap of DEGs between GBM and control samples (TCGA-GBM). **(B)** Volcano plot and heatmap of DEGs between TMZ-resistant and TMZ-sensitive samples (GSE100736). **(C)** Venn diagrams of the multi-dimensional intersection strategy, showing overlap among TCGA-GBM, GSE100736, and key SM-associated genes for upregulated **(C**(a)**)** and downregulated **(C**(b)**)** subsets. **(D, E)** GO biological processes **(D)** and KEGG pathways **(E)** enriched in the identified GSTR genes.

To isolate specific genes at the interface of the sphingolipid-associated molecular landscape and TMZ resistance in GBM, we performed a multi-dimensional intersection analysis. First, 9,687 DEGs distinguishing GBM from control samples were identified in TCGA-GBM and designated as DEGs #2 (**Figure 3A**), while 8,272 DEGs distinguishing TMZ-resistant from TMZ-sensitive samples were identified in GSE100736 and designated as DEGs #3 (**Figure 3B**). We prioritized 2,010 genes exhibiting functionally concordant trends, specifically targeting those acting as potential drivers of both tumorigenesis and chemoresistance (simultaneously upregulated) or as dual suppressors (simultaneously downregulated) across both datasets. These candidates were subsequently intersected with the SM-associated DEGs (DEGs #1) and key WGCNA module genes defined in our preceding analysis. This rigorous screening converged on 95 GSTR genes (**Figure 3C**). Functional enrichment analysis indicated that these GSTR genes are heavily implicated in immune-related GO terms, such as lymphocyte-mediated immunity, humoral immune response, and regulation of innate immune response (**Figure 3D**). KEGG pathway analysis highlighted their enrichment in the intestinal immune network for IgA production, DNA replication, cell cycle, galactose metabolism, and NOD-like receptor signaling pathways (**Figure 3E**).

### 3.4 Construction and validation of a reliable six-gene prognostic model

**Figure 4:**
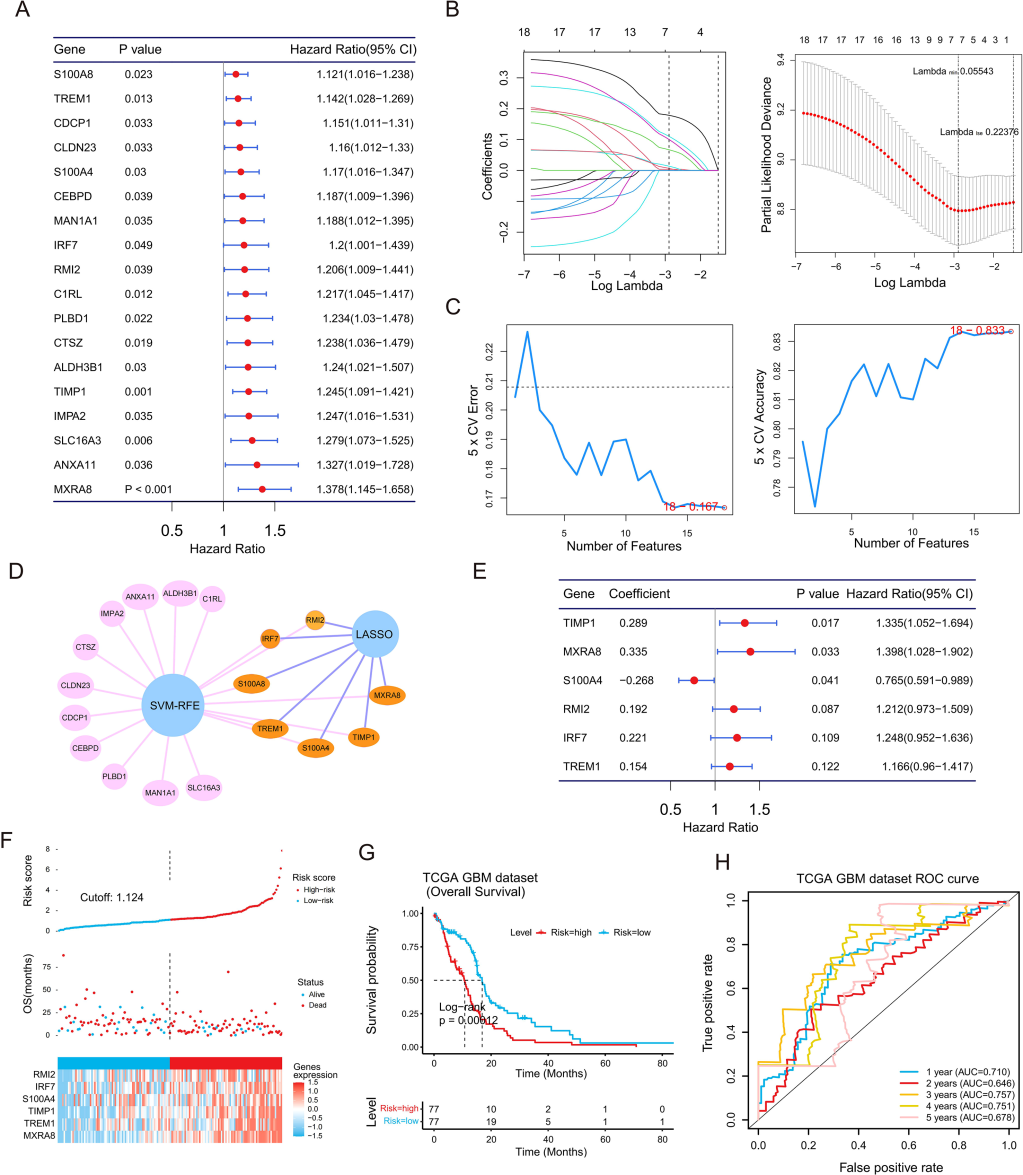
Construction of the six-gene prognostic model. **(A)** Forest plot of 18 candidate genes identified by univariate Cox regression. **(B)** LASSO Cox regression showing coefficient profiles (left) and cross-validation for optimal parameter selection (right). **(C)** SVM-RFE algorithm ranking gene importance to minimize error rate. **(D)** Intersection of candidates selected by LASSO and SVM-RFE. **(E)** Multivariate Cox regression establishing the final six-gene model. **(F)** Risk score distribution (top), survival status (middle), and expression heatmap (bottom) in the TCGA-GBM cohort. **(G)** K-M curves of OS for high-risk vs. low-risk groups. **(H)** Time-dependent ROC curves for 1- to 5-year survival predictions.

To optimize feature selection for prognostic modeling, univariate Cox regression analysis of the GSTR genes identified 18 candidates with prognostic significance (P < 0.05) (**Figure 4A**). LASSO regression refined this list to 7 genes (**Figure 4B**), while SVM-RFE independently identified 18 genes (**Figure 4C**). The intersection of these two methods prioritized 7 potential candidates (**Figure 4D**). Subsequent multivariate Cox regression analysis further refined this panel by excluding S100A8, ultimately establishing a reliable six-gene prognostic model comprising MXRA8, TIMP1, TREM1, S100A4, RMI2, and IRF7 (**Figure 4E**). Preliminary functional annotation of these signature genes indicates their distribution across three critical functional axes associated with tumor progression: MXRA8, TIMP1, and S100A4 are primarily linked to ECM remodeling; TREM1 and IRF7 are associated with inflammatory signaling; while RMI2 is involved in DNA damage repair. The expression profiles of these genes in GBM and TMZ-resistant samples are shown in **Supplementary Figure 1F**, with significant differential expression observed between tumor and normal tissues (**Supplementary Figure 1G**). The risk score distribution indicated that deceased patients were concentrated in the high-risk group (**Figure 4F**). K-M analysis confirmed that the high-risk group had significantly worse overall survival than the low-risk group (**Figure 4G**). The model demonstrated high predictive accuracy, with area under the curve (AUC) values for 1-, 2-, 3-, 4-, and 5-year survival all exceeding 0.6 (**Figure 4H**). These results were successfully validated in an independent external validation cohort (**Supplementary Figures 1H**–**J**). Furthermore, the prognostic risk score was positively correlated with the expression of the classic TMZ resistance marker MGMT and was higher in tumors with unmethylated MGMT promoters (**Supplementary Figure 2A**).

### 3.5 Independent prognostic value and association with immune pathways

**Figure 5:**
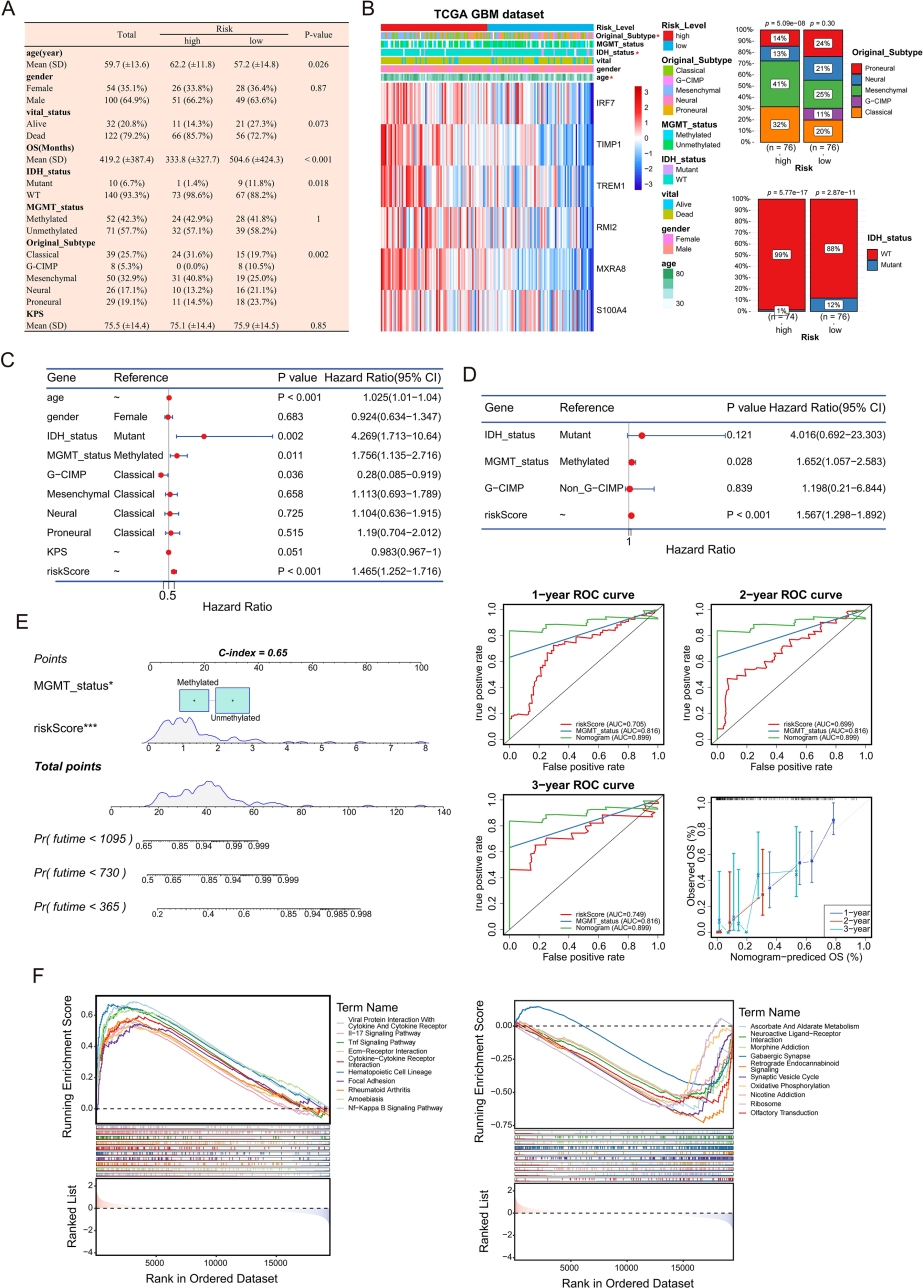
Independent prognostic value, clinical and biological relevance of the risk model. **(A, B)** Heatmap and bar plots illustrating the association among risk stratification, clinicopathological features **(A)**, and model gene expression **(B)**. **(C, D)** Univariate **(C)** and multivariate **(D)** Cox regression analyses identifying the risk score and MGMT status as independent prognostic factors. **(E)** Nomogram for predicting 1-, 2-, and 3-year OS (left), validated by calibration plots and time-dependent ROC curves (right). **(F)** GSEA plots visualizing signaling pathways significantly correlated with the risk score.

We further evaluated the independent prognostic value of the model and characterized its clinical and biological relevance. Clinical characteristics and prognostic gene distribution differed significantly between high- and low-risk groups (**Figures 5A, B**). The risk score emerged as an independent prognostic factor alongside MGMT promoter status in both univariate and multivariate Cox regression analyses (**Figures 5C, D**). A nomogram incorporating these factors showed superior predictive performance, validated by calibration plots and ROC curves (**Figure 5E**). Biologically, GSEA revealed a stark contrast in functional states. The high-risk score was positively correlated with immune responses and stromal remodeling pathways, including IL-17, TNF, NF-kappaB signaling, focal adhesion, and ECM-receptor interaction. Conversely, it was negatively correlated with essential metabolic processes and differentiated neuronal signatures, such as oxidative phosphorylation, synaptic vesicle cycle, and GABAergic synapse (**Figure 5F**). Collectively, these findings intimate that the risk signature captures a fundamental phenotypic shift from metabolic and neuronal homeostasis toward an aggressive, inflammatory, and stroma-rich malignancy.

### 3.6 Immune exclusion and exhaustion characterize the high-risk TME

**Figure 6:**
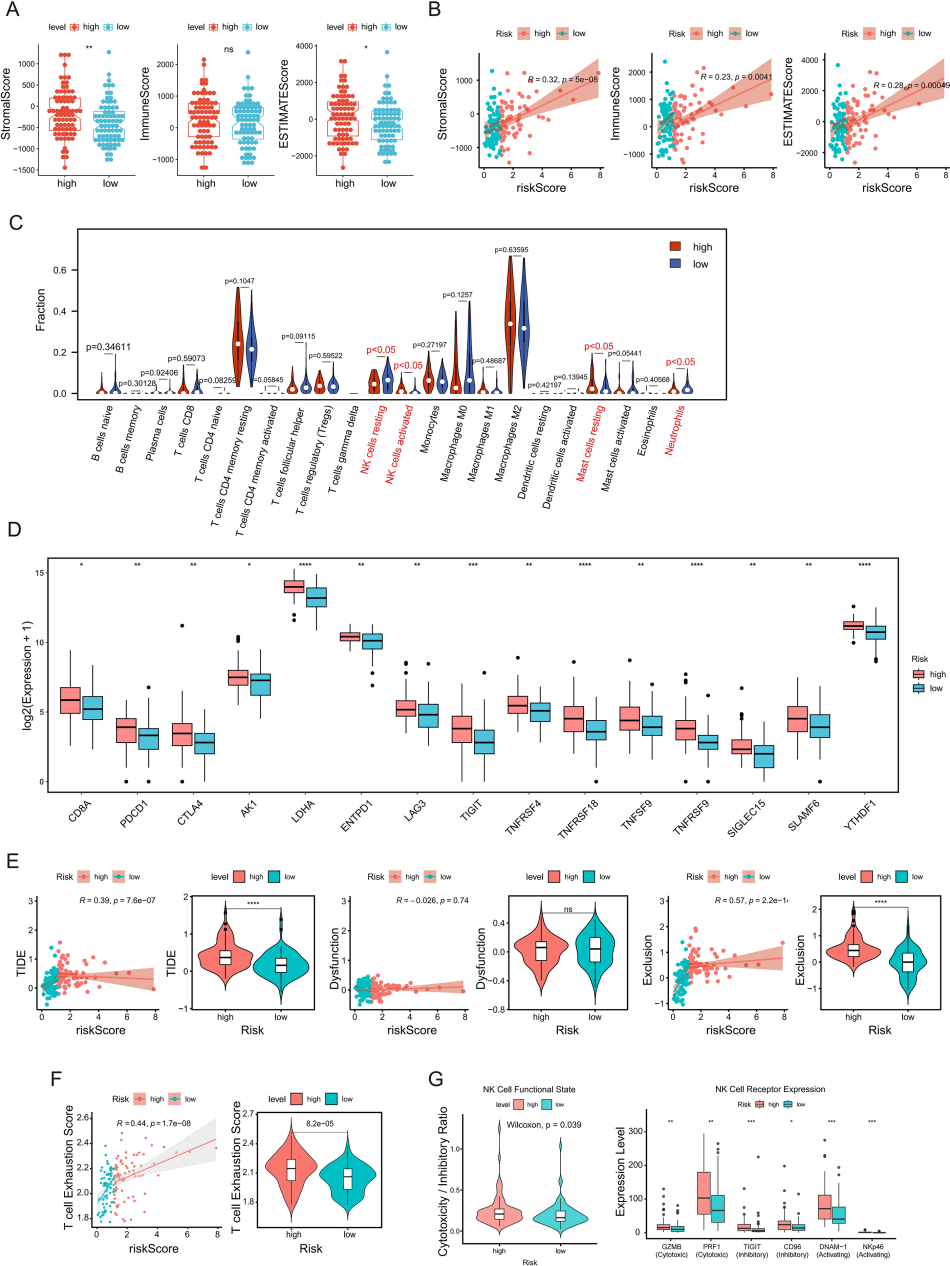
Immune microenvironment patterns associated with the sphingolipid metabolism–related risk score in TCGA−GBM. **(A)** Comparison of Stromal, Immune, and ESTIMATE scores between risk groups. **(B)** Associations between the risk score and selected TME signature scores. **(C)** Violin plots of differential immune cell infiltration (CIBERSORT). **(D)** Expression of immune checkpoint and T-cell exhaustion–related genes. **(E)** TIDE-derived TIDE, Dysfunction, and Exclusion scores across risk groups. **(F)** ssGSEA-based T-cell exhaustion score and its association with risk scores. **(G)** NK functional state assessed by the cytotoxicity-to-inhibitory ratio and expression of key NK effector molecules and receptors. * P < 0.05; ** P < 0.01; *** P < 0.001; **** P < 0.0001.

To elucidate the immune-escape mechanisms captured by the sphingolipid metabolism–related risk score, we dissected the TME landscape. The high-risk group exhibited significantly elevated Stromal and ESTIMATE scores, which showed positive correlations with the risk score (**Figures 6A, B**), indicating the presence of a dense stromal barrier characteristic of immune exclusion. CIBERSORT analysis revealed distinct immune alterations, including differential infiltration of resting NK cells, activated NK cells, resting mast cells, and neutrophils (**Figure 6C**). The high-risk group displayed upregulated expression of exhaustion-associated and checkpoint genes, including key inhibitory receptors (PDCD1, CTLA4, LAG3) and co-stimulatory molecules (TNFRSF9, TNFRSF18), consistent with a T-cell exhaustion phenotype (**Figure 6D**) (23). We employed TIDE to characterize the immune phenotype and distinguish tumor immune exclusion from dysfunction. The overall TIDE score and Exclusion score were significantly higher in the high-risk group, with positive correlations to the risk score, whereas the Dysfunction score did not show significant group differences (**Figure 6E**). To provide direct transcriptional measurement of T-cell exhaustion, we computed an exhaustion score using ssGSEA on exhaustion-associated gene signatures from MSigDB C7 collections. Statistical analysis confirmed a positive correlation with the risk score and significant elevation in the high-risk group (**Figure 6F**), corroborating the presence of dual immunosuppressive features within the T-cell compartment. Furthermore, functional profiling of NK cell subsets identified a paradoxical phenotype in the high-risk group. While we observed an upregulation of cytotoxic effectors (GZMB, PRF1) and activating receptors (DNAM-1, NKp46), this was accompanied by a concurrent elevation of inhibitory checkpoints (TIGIT, CD96). This distinct co-expression pattern suggests a state of “frustrated activation,” indicating that these NK cells, despite their cytotoxic potential, are progressively transitioning toward functional exhaustion under inhibitory pressure (**Figure 6G**). Collectively, these data delineate a TME defined by multifaceted immune escape mechanisms: stromal-mediated immune exclusion, intrinsic T-cell exhaustion, and an NK cell compartment that is actively transitioning toward a dysfunctional, exhausted state.

### 3.7 Distinct therapeutic sensitivities enable stratified treatment

**Figure 7:**
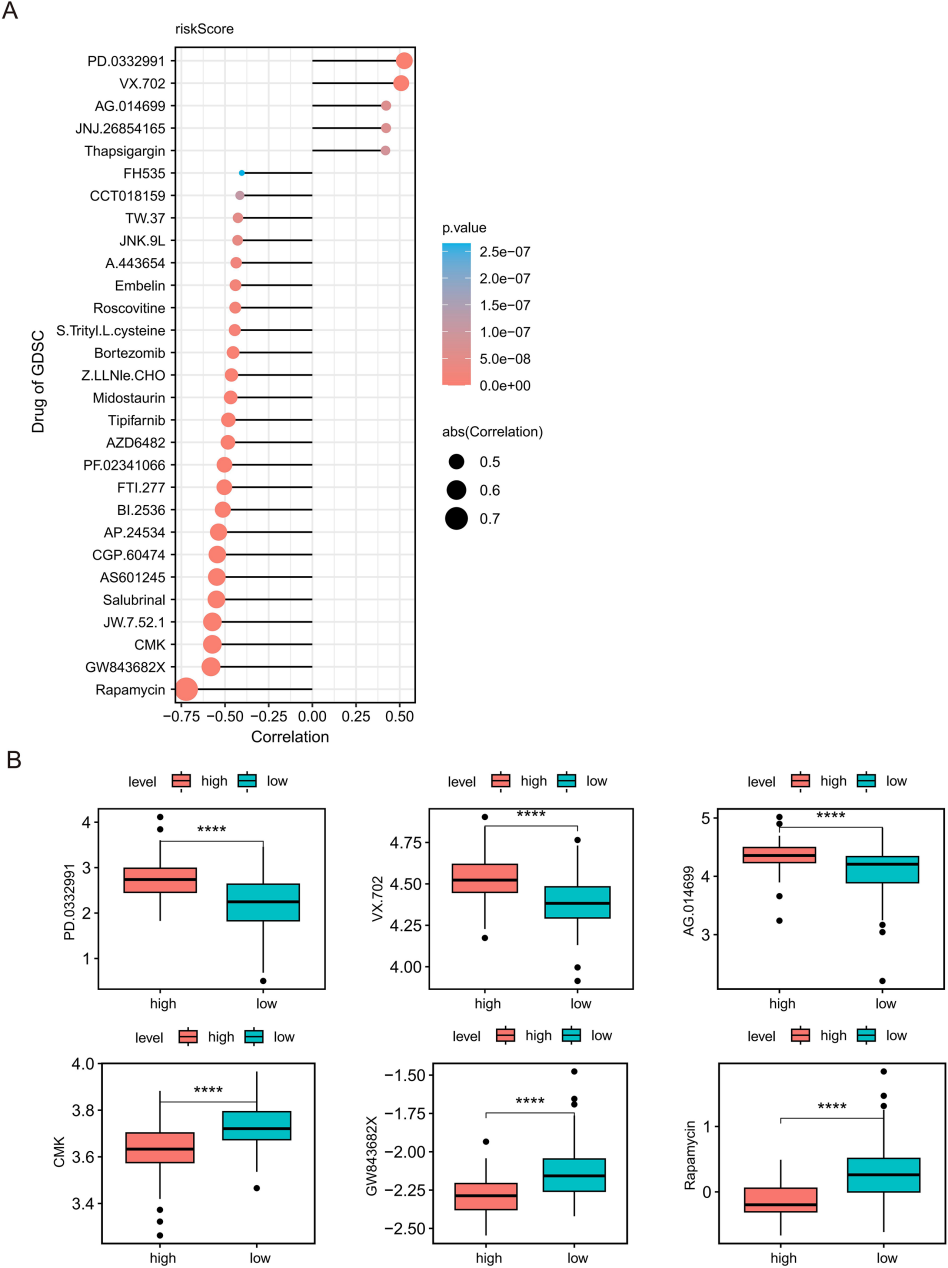
Pharmacogenomic stratification and drug sensitivity profiling. **(A)** Lollipop plot correlating risk scores with estimated IC50 values of chemotherapeutic agents (GDSC). Dot size indicates correlation strength; color indicates significance. **(B)** Box plots of estimated IC50 values for representative drugs. The high-risk group shows sensitivity to Rapamycin, CMK, and GW843682X; the low-risk group responds to PD.0332991, VX.702, and AG.014699. **** P < 0.0001.

To identify candidate therapeutic agents for stratified management, we performed a pharmacogenomic analysis using the GDSC database to predict chemotherapeutic response. We identified distinct therapeutic landscapes for different risk subgroups based on the estimated IC50 values of 138 drugs. As illustrated in **Figure 7A**, 24 agents exhibited a significant negative correlation with the risk score (P < 0.05, Cor < -0.4), while 5 agents showed a significant positive correlation (P < 0.05, Cor > 0.4).

Notably, the high-risk group demonstrated significantly lower estimated IC50 values for Rapamycin (an mTOR inhibitor), CMK (a PLK1 inhibitor), and GW843682X compared to the low-risk group (**Figure 7B**). Crucially, the pronounced sensitivity to mTOR and PLK1 inhibitors aligns with reported mechanisms linking these pathways to glioma malignancy and chemoresistance (24, 25), suggesting that high-risk tumors may exhibit “metabolic addiction” to these survival axes. Conversely, the low-risk group exhibited pronounced sensitivity (lower IC50) to PD.0332991 (Palbociclib, a CDK4/6 inhibitor), VX.702, and AG.014699. This indicates a specific dependency on canonical cell cycle checkpoints, which are effectively targeted by agents such as PD.0332991 (**Figures 7A, B**). Collectively, these findings reveal distinct therapeutic vulnerabilities, providing a compelling rationale for stratified precision medicine strategies.

### 3.8 Clinical validation of the prognostic signature at transcriptional and proteomic levels

**Figure 8:**
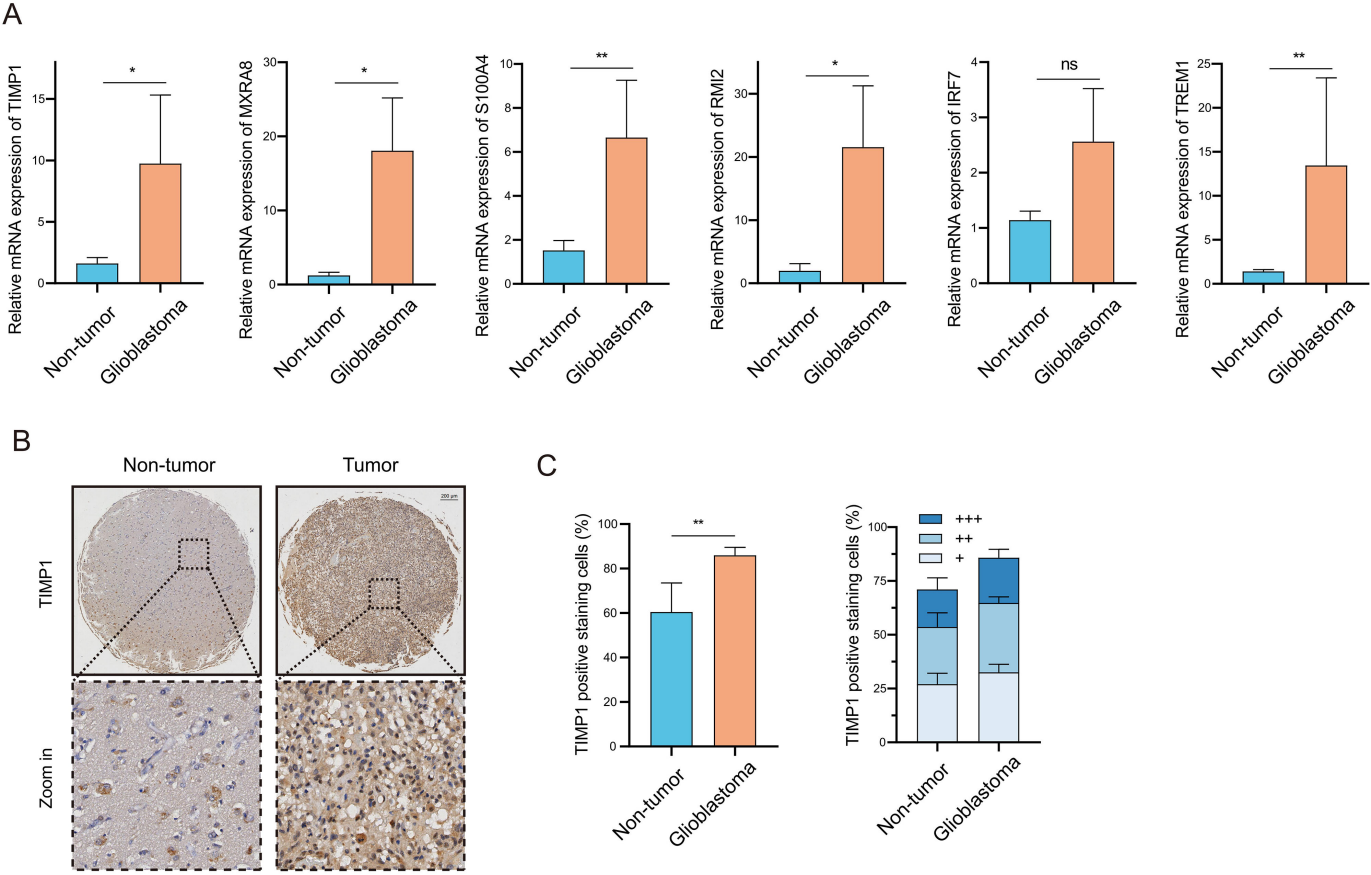
Clinical validation of the prognostic signature at transcriptional and proteomic levels. **(A)** Relative mRNA expression of five prognostic model genes (MXRA8, TIMP1, TREM1, S100A4, RMI2) in clinical GBM tissues compared to adjacent normal tissues quantified by RT-qPCR. **(B)** Representative IHC staining images of TIMP1 protein in normal brain and GBM tissues. Scale bar: 200 μm. **(C)** Quantitative analysis of TIMP1 protein expression in the glioma TMA, where +, ++, and +++ represent the proportion of cells with corresponding positive signals (weak [+], moderate [++], or strong [+++]). Data are presented as box plots. * P < 0.05; ** P < 0.01.

To bridge the gap between computational predictions and clinical reality, we validated the expression of model genes using patient-derived specimens. RT-qPCR analysis revealed a pronounced upregulation of MXRA8, TIMP1, TREM1, S100A4, and RMI2 in GBM tissues compared to adjacent non-tumor tissues, consistent with the expression trends identified in public databases (**Figures 8A**, **Supplementary Figure 1G**). To further corroborate these findings at the translational level, we prioritized TIMP1 for validation of protein expression. IHC staining of TMA confirmed that TIMP1 protein was aberrantly overexpressed in GBM relative to normal brain tissues (**Figures 8B, C**). This dual-level validation reinforces the reliability of TIMP1 as a representative biomarker of our model. While protein-level verification of the remaining genes warrants further investigation in future studies, our findings confirm that the key bioinformatic signatures are faithfully recapitulated in the clinical setting.

### 3.9 TIMP1 orchestrates GBM malignancy and confers resistance to TMZ

**Figure 9:**
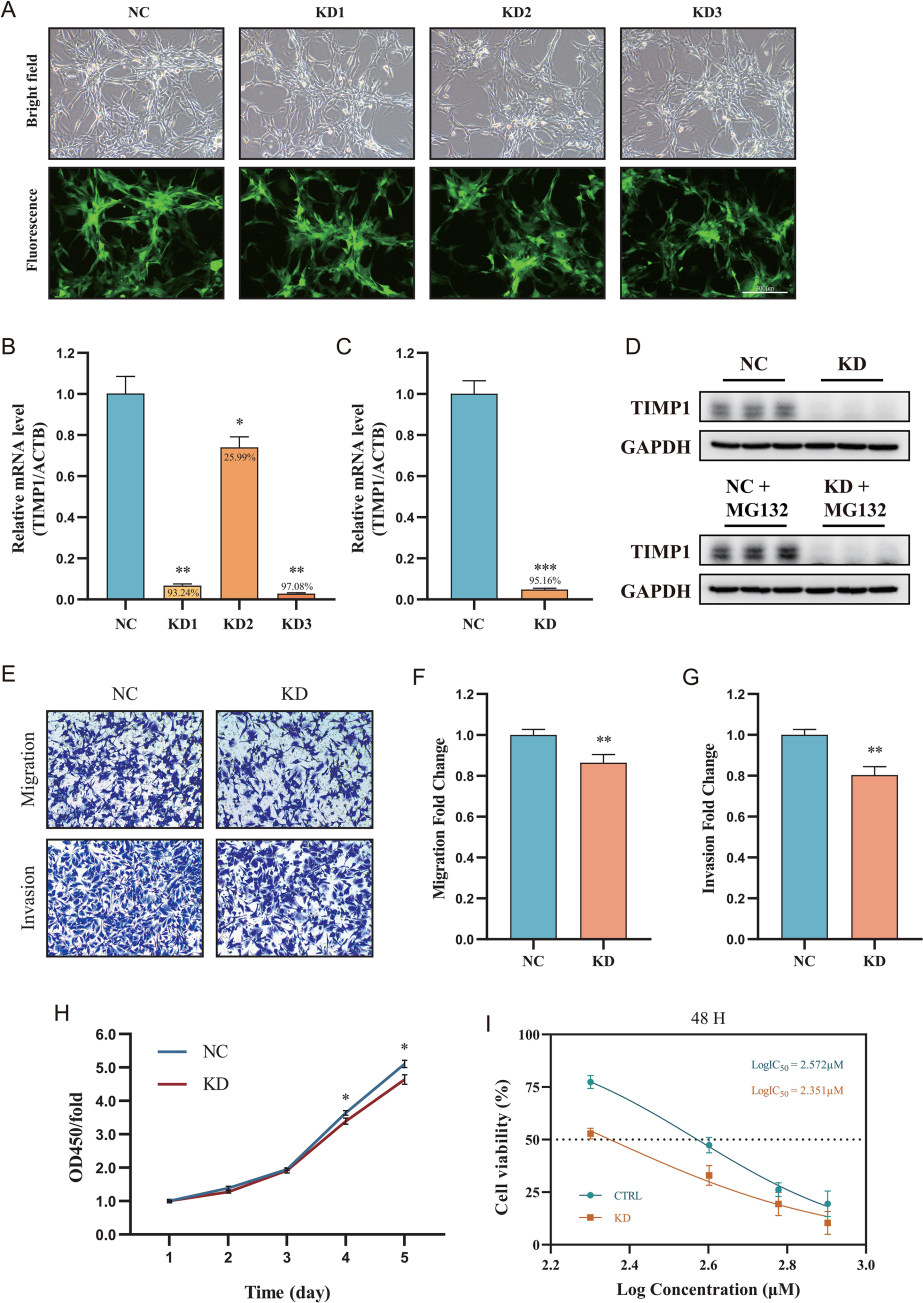
TIMP1 drives GBM malignancy and confers resistance to TMZ. **(A–D)** Verification of lentiviral-mediated TIMP1 knockdown efficiency in U87 cells, assessed by representative bright-field and fluorescence imaging **(A)**, RT-qPCR **(B, C)**, and Western blotting **(D)**. Numbers indicate the percentage of knockdown efficiency. **(E–H)** Impact of TIMP1 silencing on GBM cell phenotypes. Proliferation was evaluated via CCK-8 assays, while migration and invasion capabilities were assessed using Transwell assays. **(I)** Dose-response curves determining the IC50 values of TMZ in vector-control and TIMP1-knockdown U87 cells. * P < 0.05; ** P < 0.01; *** P < 0.001.

To decipher the functional contribution of the core gene TIMP1 to GBM progression and chemoresistance, we performed gene knockdown experiments *in vitro*. The efficacy of TIMP1 silencing was strictly verified at both mRNA and protein levels (**Figures 9A–D**). Phenotypically, depletion of TIMP1 significantly suppressed the oncogenic potential of GBM cells, as evidenced by significantly attenuated proliferation, migration, and invasion capabilities (**Figures 9E–H**). Crucially, in the context of chemotherapy, TIMP1 silencing potentiated the cytotoxic efficacy of TMZ, resulting in a marked reduction in the IC50 value (**Figure 9I**). Consistent with this, database analysis revealed that TIMP1 expression was positively correlated with the expression of the classic TMZ resistance marker MGMT and was higher in tumors with unmethylated MGMT promoters (**Supplementary Figure 2B**). These findings position TIMP1 as a critical functional node that not only drives intrinsic malignancy but also mediates resistance to TMZ therapy in GBM.

### 3.10 Proposed integrated working model: sphingolipid dysregulation and chemoresistance

**Figure 10:**
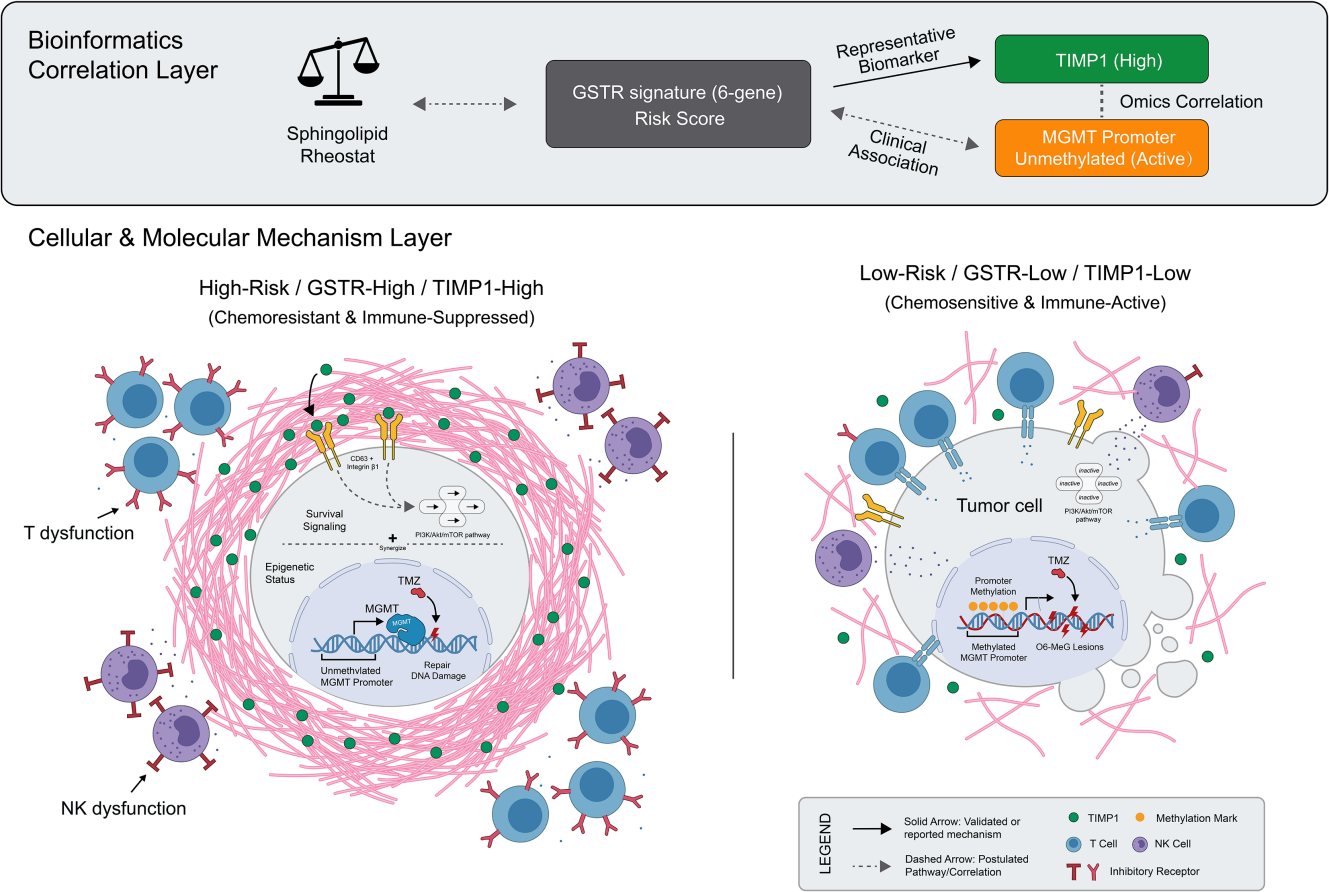
A mechanistic hypothesis of GSTR-mediated GBM malignancy. The GSTR signature reflects the dysregulated sphingolipid molecular landscape, with TIMP1 acting as a representative hub. It is hypothesized that TIMP1-mediated signaling stimulates PI3K/Akt/mTOR pathways and correlates with MGMT-mediated DNA repair to confer TMZ resistance. Concurrently, this process drives stromal remodeling and physical barrier formation, inducing an immune-excluded microenvironment characterized by T-cell and NK-cell dysfunction. (Solid arrows denote validated or reported mechanisms; dashed arrows represent postulated pathways or correlations).

Based on our integrative stratification analyses and functional validation, we propose a putative working model (**Figure 10**). In this framework, dysregulated sphingolipid metabolism is hypothesized to occupy an upstream regulatory position associated with a coordinated transcriptomic shift of the tumor microenvironment. This shift is captured by the six-gene GSTR signature, which manifests through three distinct potential functional programs: (1) ECM remodeling (potentially mediated by TIMP1, MXRA8, and S100A4) ; (2) Chronic inflammatory signaling (TREM1 (26) and IRF7 (27)) which may orchestrate a pathological environment linked to immune exhaustion and exclusion; and (3) Enhanced DNA repair capacity (likely associated with RMI2 and the TIMP1-MGMT correlation). Collectively, these processes converge to create a “protected” tumor niche that not only heightens intrinsic malignancy and aggressiveness but also safeguards tumor cells against TMZ cytotoxicity. However, it is important to note that while our findings strongly associate the GSTR signature with an immune-excluded phenotype, causal validation of this signature-driven microenvironment remodeling relies on future mechanistic studies.

## 4 Discussion

The clinical management of GBM remains deadlocked by TMZ resistance. While sphingolipid metabolism and the “sphingolipid rheostat” mechanism are increasingly recognized as crucial determinants of cell fate and therapeutic response (4), the clinical potential of these metabolic processes and their associated molecular landscape for patient stratification remains largely untapped; specifically, the comprehensive prognostic value of SMRGs and their regulatory role in TMZ sensitivity in GBM remain largely unexplored. To bridge this gap, we performed an integrated analysis of public transcriptomic datasets, unveiling a distinct set of GSTR genes that map the interplay between the sphingolipid-associated molecular landscape and chemoresistance. Our findings suggest that these biomarkers represent molecular shifts linked to sphingolipid dysregulation, capturing the downstream stromal and inflammatory remodeling that fosters therapeutic resistance. Based on this, we constructed a six-gene prognostic model, validated its prognostic performance, and characterized its association with an immune-excluded niche. Furthermore, TIMP1 was functionally validated *in vitro* as a representative driver of TMZ resistance. These findings provide a novel molecular framework for precision prognosis and offer a theoretical rationale for targeting the sphingolipid-associated axis to overcome resistance.

The six prognostic genes identified in our study can be conceptually grouped into distinct functional programs, collectively concretizing upstream sphingolipid dysregulation into a comprehensive malignant phenotype characterized by both heightened aggressiveness and therapeutic resistance. First, the synergy within the ECM remodeling axis (comprising TIMP1 (28), MXRA8 (29), and S100A4 (30)) likely extends beyond simple matrix stiffening; it is hypothesized to create a physical barrier that restricts drug penetration while providing pro-survival biomechanical cues that fuel invasion. Second, the chronic inflammatory axis (potentially driven by TREM1 (26) and IRF7 (27)) may orchestrate a pathological environment that fosters immune exhaustion and exclusion, rather than effective anti-tumor immunity. Third, the intrinsic repair axis provides a genetic defense against TMZ-induced damage, where TIMP1 likely acts in concert with MGMT and RMI2 (31) to bolster DNA repair efficiency. By integrating these multifaceted mechanisms, the GSTR signature provides a systematic framework for understanding how sphingolipid-driven molecular shifts ultimately protect tumor cells from genotoxic stress while driving their malignant progression.

To move from bioinformatic prediction to functional verification, we confirmed the upregulation of core risk genes in clinical specimens via RT-qPCR and established TIMP1 as a functional driver of chemoresistance, where its silencing in U87 cells markedly enhanced TMZ sensitivity. Mechanistically, our findings suggest a critical functional dichotomy of TIMP1 in GBM. Based on this, we postulate that beyond its canonical MMP-inhibitory role, its non-canonical engagement of the CD63/integrin β1 complex to activate a PI3K/Akt/mTOR pro−survival axis (32) exerts a more dominant influence on chemoresistance. This functional hierarchy suggests that the pro-survival signaling of TIMP1 may override its homeostatic role in matrix regulation, potentially explaining why conventional therapies targeting matrix degradation alone often fail. In turn, Akt-dependent modulation of sphingolipid metabolism may favor S1P-biased signaling over pro-apoptotic ceramide, thereby supporting resistance to alkylating damage (33). This is consistent with the enrichment of TNF and NF-κB pathways observed in high-risk tumors, and is further supported by their preferential sensitivity to Rapamycin, suggesting a dependence on mTOR-associated survival signaling. Concurrently, the elevated stromal scores and high TIDE exclusion scores point to the overarching GSTR program, where TIMP1 acts as a key functional component to orchestrate a “physical and immunological barrier.” By promoting ECM remodeling, this GSTR-associated axis facilitates a state of spatial exclusion rather than simple matrix stiffening, reducing effective TMZ distribution and contributing to the maintenance of an immune-evasive niche that protects residual tumor clones (34–36). Collectively, integrating our results with existing literature, these findings highlight the postulated TIMP1–PI3K/Akt–sphingolipid axis as a critical vulnerability, where TIMP1 prioritizes malignant cell survival over normal tissue architecture.

At a systemic level, our pathway enrichment analysis provided a broader molecular blueprint of the high-risk phenotype. Specifically, the risk score was positively associated with inflammatory and stromal remodeling cascades, such as TNF and NF-kappaB signaling, alongside ECM-receptor interaction. Rather than implying a generic immunosuppressive state, these cascades align with an immune-evasive context where chronic inflammation and matrix remodeling jointly promote therapeutic resistance. These pathways facilitate stromal-mediated immune exclusion by limiting effective immune-cell trafficking into the tumor parenchyma while simultaneously driving functional exhaustion of the effector compartment that manages to infiltrate under persistent antigenic pressure. Conversely, the risk score was negatively correlated with energy metabolism (oxidative phosphorylation) and neuronal signaling (neuroactive ligand-receptor interaction, GABAergic synapse). This points to profound metabolic reprogramming and a simultaneous loss of differentiated neuronal identity. Our findings suggest that the aberrant expression of SMRGs serves as a molecular reflection of a dysregulated sphingolipid rheostat, which we propose orchestrates this observed transition from a metabolically active state to an aggressive, inflammatory malignancy. Literature evidence confirms extensive crosstalk between sphingolipid signaling and these pathways; for example, ceramide can modulate NF-kappaB activation, while S1P is a potent inducer of inflammation and cell survival (37, 38). We speculate that this transcriptomic transition represents a fundamental “identity crisis” in high-risk GBM cells, where they jettison energy-expensive neuronal differentiation in favor of a robust although chaotic inflammatory survival program. This leads us to hypothesize that such strategic dedifferentiation is governed by the sphingolipid rheostat acting as a master metabolic switch to redefine the biological priorities of the tumor. In this context, we suggest that the tumor may prioritize the sacrifice of functional homeostasis to build a resilient and drug-resistant niche.

By integrating ESTIMATE, CIBERSORT, TIDE, and ssGSEA alongside immune checkpoint expression profiles, we systematically characterized the TME landscape. High-risk patients exhibited significantly higher Stromal and ESTIMATE scores, indicating a complex, stroma-rich microenvironment that may act as a physical barrier and source of inhibitory factors to drive tumor progression. Notably, the high-risk group concurrently displayed elevated expression of key immune checkpoints (e.g., PDCD1, CTLA4, LAG3) alongside effector markers like CD8A. While this pattern typically implies T-cell exhaustion, the high stromal and exclusion scores observed in our model suggest that this dysfunction is likely intertwined with a more complex spatial exclusion mechanism. This indicates that the functional impairment of infiltrating lymphocytes is not merely a consequence of chronic signaling, but is reinforced by GSTR-associated ECM remodeling, where TIMP1 acts as a key component to form a physical barrier restricting effector cell trafficking. Such a structural-biochemical synergy suggests that in high-risk GBM, reversing effector exhaustion via PD-1 inhibitors alone may be insufficient unless the stromal-mediated “physical exclusion” is simultaneously targeted to facilitate therapeutic penetration. Furthermore, CIBERSORT analysis revealed significant differences in NK cell subset distributions (39, 40). The specific “frustrated activation” phenotype (co-expression of cytotoxic and inhibitory markers) adds another layer to this multifaceted immune escape mechanism. Collectively, as summarized in our integrated mechanism (**Figure 10**), these data depict a TME defined by the synergy between stromal-mediated immune exclusion and intrinsic effector cell exhaustion, guiding the development of combinatorial immunotherapeutic strategies.

Given the robust mechanistic basis linking sphingolipid dysregulation to therapeutic resistance, establishing a reliable prognostic biomarker for clinical translation is essential. Consistently, our multivariate Cox analysis confirmed that the GSTR-based risk score is an independent predictor of GBM prognosis, alongside the established MGMT promoter methylation status (41). Notably, the risk score retained its prognostic value even when adjusted for MGMT status, suggesting that the GSTR signature captures a multidimensional resistance ecosystem encompassing stromal remodeling, immune evasion, and auxiliary DNA repair, which complements MGMT-mediated mechanisms. Consequently, this score may explain treatment failure in patients who theoretically possess a favorable MGMT status but succumb to sphingolipid-driven malignancy. Furthermore, the construction of a nomogram integrating the risk score with clinical factors further demonstrated the model’s translational potential, with calibration and ROC curves confirming its accuracy and reliability (42). This model represents a potential tool for mechanism-based stratification. However, translating this statistical significance into clinical utility will require validating whether this risk score can guide therapeutic decision-making beyond standard-of-care, specifically in identifying candidates for emerging metabolic or stromal-targeted interventions.

Our pharmacogenomic profiling identified potential therapeutic agents associated with the risk score, providing valuable avenues for drug repurposing and combination strategies, offering potential directions to overcome TMZ resistance. Our analysis revealed significant drug sensitivity heterogeneity between high- and low-risk GBM subgroups. Specifically, 24 agents, including Rapamycin and CMK, were negatively correlated with the risk score, indicating higher sensitivity in the high-risk group. This suggests that these agents may be more effective for patients with high-risk tumors. Previous studies have established that S1P is a potent activator of mTOR signaling, which promotes cell cycle progression and blocks TMZ-induced apoptosis, thereby conferring chemoresistance (43–45). Our data align seamlessly with this mechanism: the high-risk GBM phenotype, often associated with a dysregulated sphingolipid rheostat, exhibits pronounced sensitivity to Rapamycin, suggesting these tumors have developed a ‘metabolic addiction’ to the S1P-mTOR survival axis. In this context, Rapamycin could precisely intercept this survival signal, dismantling the resistance shield against TMZ. Conversely, five agents, including PD.0332991 and VX.702, correlated positively with the risk score, demonstrating higher sensitivity in the low-risk group. While p38 MAPK activation (targeted by VX.702) has been implicated in glioma chemoresistance (46) and ceramide-induced apoptosis (47), its specific role in the low-risk context warrants further investigation. These findings suggest that incorporating sphingolipid reprogramming into therapeutic considerations may offer a novel entry point for overcoming TMZ resistance and potentially achieving stratified precision treatment.

Several limitations should be acknowledged. First, while our functional assays established TIMP1 as a driver of chemoresistance, expanding validation across diverse genetic backgrounds, including patient-derived primary cells and animal xenograft models, will be essential to fully capture GBM heterogeneity and systemic therapeutic responses. Second, our identified gene signature primarily reflects the downstream molecular landscape associated with sphingolipid dysregulation; elucidating the precise dynamics of sphingolipid metabolic pathways will require further metabolomic profiling. Third, although robust in retrospective cohorts, the clinical utility of our risk model awaits validation in large-scale prospective studies. Additionally, the initial identification of TMZ resistance-associated genes relied on a dataset with limited sample size, which may introduce some uncertainty. Finally, emerging spatial and single-cell technologies could further resolve the complex immune exclusion patterns identified here. Despite these constraints, our study provides a compelling molecular framework linking the sphingolipid-associated landscape to TMZ resistance, laying a solid foundation for precision intervention.

In conclusion, through comprehensive bioinformatics analysis and systematic experimental validation, we identified a multidimensional GSTR signature that highly mirrors the sphingolipid-associated molecular landscape and TMZ resistance mechanisms. We constructed a potent risk model that not only independently predicts prognosis but also encapsulates the complex interplay between metabolic dysregulation and stromal immune exclusion. Clinical validation confirmed the upregulation of core genes, and functional experiments established TIMP1 as a key driver of malignancy and chemoresistance, providing mechanistic support for the hypothesized “physical-immunological barrier.” These findings provide new tools for precision prognostic stratification, specifically complementing established MGMT testing, and offer a solid theoretical and experimental basis for developing stratified, combination therapies targeting the sphingolipid-associated axis to overcome TMZ resistance. Future work will focus on elucidating the detailed molecular mechanisms and validating the clinical utility of this model in prospective cohorts. We remain committed to dissecting the sphingolipid network to address the therapeutic bottlenecks in GBM.

## Data Availability

The datasets presented in this study can be found in online repositories. The names of the repository/repositories and accession number(s) can be found in the article/**Supplementary Material**.

## Supplementary material

Supplementary Figure 1 Quality control and external validation. **(A)** scRNA-seq quality metrics (RNA count, feature count, mitochondrial percentage). **(B, C)** Identification of highly variable genes **(B)** and PCA dimensionality reduction **(C)**. **(D)** Expression of canonical markers for cell type annotation. **(E)** K-M analysis of High vs. Low SM groups in GSE74187. **(F)** Expression profiles of prognostic model genes in GBM cohorts and TMZ-sensitivity datasets. **(G)** Differential expression of model genes between GBM tissues (TCGA) and normal brain tissues (TCGA and GTEx). **(H)** Risk score analysis in the CGGA-325 validation cohort. **(I)** K-M survival curves for the CGGA-325 cohort. **(J)** Time-dependent ROC curves for the validation cohort.

Supplementary Figure 2 Association of the prognostic risk signature and TIMP1 with MGMT status in the TCGA-GBM cohort. **(A)** Association of the risk score with MGMT expression and promoter methylation. **(B)** Association of TIMP1 expression with MGMT expression and promoter methylation.
